# Effect of 1‐ and 2‐Month High‐Dose Alpha‐Linolenic Acid Treatment on ^13^C‐Labeled Alpha‐Linolenic Acid Incorporation and Conversion in Healthy Subjects

**DOI:** 10.1002/mnfr.201800271

**Published:** 2018-09-19

**Authors:** Marc Pignitter, Michael Lindenmeier, Gaby Andersen, Cornelia Herrfurth, Christopher Beermann, Joachim J. Schmitt, Ivo Feussner, Martin Fulda, Veronika Somoza

**Affiliations:** ^1^ Department of Physiological Chemistry, Faculty of Chemistry University of Vienna Austria; ^2^ German Research Center of Food Chemistry Freising Germany; ^3^ Department for Plant Biochemistry, Albrecht‐von‐Haller‐Institute for Plant Sciences Georg‐August‐University Goettingen Germany; ^4^ Department of Food Technology University of Applied Sciences Fulda Germany

**Keywords:** ALA conversion, compartment model, LDL, linseed oil, omega‐3 fatty acids

## Abstract

**Scope:**

The study aims at identifying 1) the most sensitive compartment among plasma phospholipids, erythrocytes, and LDL for studying alpha‐linolenic acid (ALA) conversion, and 2) whether ALA incorporation and conversion is saturable after administration of ^13^C‐labeled ALA‐rich linseed oil (LO). The effect of a daily intake of 7 g nonlabeled LO (>43% w/w ALA) for 1 month after bolus administration of 7 g ^13^C‐labeled LO on day 1, and for 2 months after bolus administration of 7 g ^13^C‐labeled LO on day 1 and day 29 on ^13^C‐ALA incorporation and conversion into its higher homologs is investigated in healthy volunteers.

**Methods and results:**

Incorporation and conversion of LO‐derived ^13^C‐labeled ALA is quantified by applying compartmental modeling. After bolus administration, a fractional conversion of approximately 30% from ^13^C‐ALA to ^13^C‐DHA is calculated as reflected by the LDL compartment. Treatment with LO for 8 weeks induces a mean reduction of ^13^C‐ALA conversion to ^13^C‐DHA by 48% as reflected by the LDL compartment, and a mean reduction of the ^13^C‐ALA incorporation into LDL by 46%.

**Conclusion:**

A 2‐month dietary intake of a high dose of LO is sufficient to reach saturation of ALA incorporation into LDL particles, which are responsible for ALA distribution in the body.

## 1 Introduction

Dietary intake of long‐chain (≥20 carbon atoms) n‐3 polyunsaturated fatty acids (n‐3 PUFA) has been inversely associated with the risk of myocardial infarction and sudden cardiac death in coronary heart disease patients.^1^ Furthermore, there is a strong correlation between coronary heart disease risk and triglyceride levels in plasma^2^ for which a reduction by the dietary n‐3 fatty acids, eicosapentaenoic acid (EPA) and docosahexaenoic acid (DHA), has been demonstrated.^3^ n‐3 PUFA have also been considered to promote anti‐inflammatory actions^4^ and be beneficial for early neurological development and visual function.^5^, ^6^

Dietary sources of EPA and DHA are limited and recommended daily intakes are rarely met by the general population, e.g., the daily recommended intake of EPA and DHA is met in only 26% of 17 European countries, as reviewed by Sioen et al. against recommendations of the European Food Safety Authority.^7^ The dominant n‐3 fatty acid in the Western diet is ALA, for which major dietary sources are green leaves, canola oil, and linseed oil (LO). Especially LO is known to be rich in ALA, with an average amount of approximately 50 g ALA per 100 g oil.^8^ EPA and DHA are found in fish and fish oil, reaching concentrations of 0.1 to 5.3 g n‐3 PUFA per 100 g seafood.^9^ However, the worldwide fish supply is limited due to overfishing, whereas fish consumption in some areas is seen critically due to persistent contamination with heavy metals and organic pollutants.^10^ Dietary ALA‐rich vegetable oils may serve as alternative source not only to increase ALA incorporation into cell membranes but also to favor conversion to its long‐chain homologues, EPA, and DHA.

A pivotal factor of ALA conversion and further metabolization is its bioavailability. By quantifying n‐3 PUFA incorporation, it was shown that the bioavailability of dietary n‐3 PUFA is about 50% higher compared to that of their corresponding triglycerides, while their corresponding ethyl esters show a bioavailability, which is 50% lower than their triglycerides.^11^ So far, conversion of stable isotope labeled ALA into EPA and DHA in humans has only been studied using either the free fatty acid^12^, ^13^, ^14^ or its ethyl esters,^5^, ^15^, ^16^ whereas human intervention studies investigating the incorporation and conversion of ALA by administration of ^13^C‐labeled ALA triglycerides are lacking.

After absorption, n‐3 PUFA may follow different pathways.^17^ First, they may be converted into their long‐chain homologues, or, second, stored in, e.g., adipose tissue, phospholipids, and cellular membranes. ALA has been demonstrated to be incorporated into adipose tissue, where it represents approximately 0.7% of total fatty acids in neutral lipids,^18^, ^19^ indicating a saturation of ALA incorporation.^20^ Third, n‐3 PUFA may be subjected to oxidation, resulting in the formation of eicosanoids via the cyclooxygenase‐ or lipoxygenase‐catalyzed reactions,^21^ or ATP via catabolic pathways through β‐oxidation.^22^ After administration of standardized meal to six healthy women for 8 days, more than 60% of dietary ALA was used as energy substrate due to ß‐oxidation.^22^

ALA has also been shown to be converted into its elongation products, EPA, DPA (docosapentaenoic acid), and DHA,^15^, ^23^, ^24^ with conversion rates determined by the absolute amounts of ALA and the n‐6/n‐3 fatty acid ratio in the diet.^14^, ^25^ According to Kris‐Etherton et al.,^26^ a typical diet in the United States provides an n‐6/n‐3 fatty acid ratio of approximately 10:1, while a lower ratio of 5:1 is recommended. In general, a diet with a low n‐6/n‐3 fatty acid ratio and administration of high amounts of ALA are considered to promote ALA elongation. However, due to the typical Western diet with a high n‐6/n‐3 fatty acid ratio, ALA conversion to DHA has been shown to be rather low, ranging from 0% to 3.8% in humans.^15^, ^24^, ^27^, ^28^, ^29^, ^30^, ^31^ Quantification of ALA conversion rates to long‐chain homologues has been performed in tracer studies applying multicompartmental modeling.^14^, ^15^, ^24^, ^32^ Most of the studies that aimed at quantifying ALA conversion by compartmental modeling studied plasma lipids and erythrocytes to reflect hepatic ALA conversion.^14^, ^15^, ^24^, ^32^, ^33^ In a human intervention study with eight healthy subjects receiving a controlled beef‐based diet with an n‐6/n‐3 fatty acid ratio of approximately 7:1 for 21 days, the fractional ALA conversion to EPA yielded 0.2% in total plasma lipids.^15^ In contrast to this rather low conversion of ALA to EPA reported earlier, a percentage fractional transfer of 99.8% was achieved in plasma phospholipids of 29 healthy subjects on a diet with an n‐6/n‐3 fatty acid ratio of 19:1.^24^ Although a diet with a high n‐6/n‐3 fatty acid ratio was consumed, ALA was almost completely converted to EPA, suggesting that isolated plasma phospholipids show higher fractional conversion of ALA to EPA compared to the total plasma lipids. Thus, for studying ALA conversion into higher homologues, a compartment that more reliably reflects hepatic conversion of ALA than the plasma needs to be identified.

We aimed at examining the compartments plasma phospholipids, erythrocytes, and LDL, for their suitability to reflect ALA incorporation and conversion to its metabolites EPA, DPA, and DHA. LDL as most sensitive compartment was chosen for quantification of ^13^C‐ALA incorporation and elongation to investigate whether ALA incorporation and conversion is saturable. The effect of a 1‐ and 2‐month dietary intake of LO, which was rich in ALA and showed a low n‐6/n‐3 fatty acid ratio, on ^13^C‐ALA incorporation and conversion after bolus administration of a ^13^C‐lableled LO was investigated by means of compartmental modeling. ^13^C‐labeled ALA bound to glycerol backbone in a food matrix was used to study the incorporation and conversion of ALA in vivo. For the first time, quantification of ^13^C‐ALA incorporation and conversion was performed by administration of a ^13^C‐labeled LO.

## 2 Experimental Section

### 2.1 Subjects

Twelve healthy male volunteers aged 20–40 years were recruited and underwent medical examinations for study participation. This sample size resulted from a statistical power calculation based on our previous study, where administration of 4.4 g ALA per day increased LDL EPA contents by 36% (alpha = 0.05; power = 80%, anticipated dropout rate 20%).^31^, ^32^ Subjects were screened for their eating behavior (dietary questionnaire) and their blood lipid status. Inclusion criteria were triglyceride concentrations of ≤2.26 mmol L^–1^, total cholesterol concentrations of <5.69 mmol L^–1^, LDL cholesterol concentrations of <4.14 mmol L^–1^, HDL cholesterol concentrations of 0.91–1.55 mmol L^–1^, and a BMI of <28 kg m^–2^. Ten subjects fulfilled the criteria and were eligible to participate in the study. The subjects had a mean BMI of 24.8 ± 2.9 kg m^–2^. They had mean ± SD triglyceride plasma concentrations of 0.89 ± 0.51 mmol L^–1^, total cholesterol concentrations of 4.15 ± 0.68 mmol L^–1^, LDL cholesterol concentrations of 2.54 ± 0.52 mmol L^–1^, and HDL cholesterol concentrations of 1.40 ± 0.18 mmol L^–1^. Participants were nonsmokers and did not use medication. The study was conducted in accordance with the Declaration of Helsinki, and the study protocol was approved by the Ethics Committee of the Technical University of Munich, project number 1737/07. Each study subject provided their written informed consent prior to the intervention.

### 2.2 Dietary Protocol and Study Design

The human intervention started with a 4‐week washout period, which was followed by two interventions, each comprising 4 weeks of LO administration of 40 g d^–1^ (= 19.7 g ALA d^–1^). The amount of ALA‐containing LO administered was chosen according to a previous study design where ALA incorporation and elongation was studied in healthy subjects after administration of canola oil‐derived, nonlabeled ALA incorporated into spreads and margarines.^31^, ^32^

The study oils were administered after blending into curd cheese or vegetable juice. The linseed variety Lirina for the oil production was provided by NPZ, the Norddeutsche Pflanzenzucht, Hohenlieth, Germany and DSV, the Deutsche Saatveredelung AG, Lippstadt, Germany. The seeds were cold‐pressed by PPM, the Pilot Pflanzenöltechnologie Magdeburg e.V., Magdeburg, Germany. For the production of the ^13^C‐labeled LO, 120 flax plants were exposed to ^13^C‐CO_2_. Plants were grown in soil in a growth chamber at 24 °C with a 16 h day:8 h night cycle, and at 60% relative humidity. Plants were exposed to ^13^C‐CO_2_ 16 days after onset of the flowering period for eight consecutive days during the light period. The concentration of total CO_2_ was controlled and adjusted to a concentration of about 400 ppm by supplying an average of 6.5 L ^13^C‐labeled CO_2_ per hour. After manual harvesting of flax seeds, they underwent cold pressing and purification with a yield of 140 g of LO. GC/MS analyses^34^ revealed the proportion of ^13^C‐labeling of ALA in flax seeds. More than 50% of total ALA content in the flax seeds was labeled at least two carbons (Figure S1, Supporting Information). Besides investigating the labeling efficacy, the fatty acid composition of the labeled and native LO was determined by high resolution‐GC/MS (**Table** 1).^35^ The carbon isotope discrimination is a well‐known phenomenon caused by differences in diffusivities in the air and by discriminating enzymes in the plant.^36^

**Table 1:** Fatty acid composition (% based on total fatty acids) of the native and the ^13^C‐labeled study oil ± SEM

			[%]			
Fatty acid	16:0	18:0	18:1	18:2	18:3	n‐6/n‐3
Native	5.30 ± 0.01	4.30 ± 0.01	21.0 ± 0.01	20.0 ± 0.01	49.0 ± 0.01	0.41
Labeled	5.60 ± 0.01	2.50 ± 0.01	37.0 ± 0.01	12.0 ± 0.01	43.0 ± 0.01	0.28

**Figure 1:**
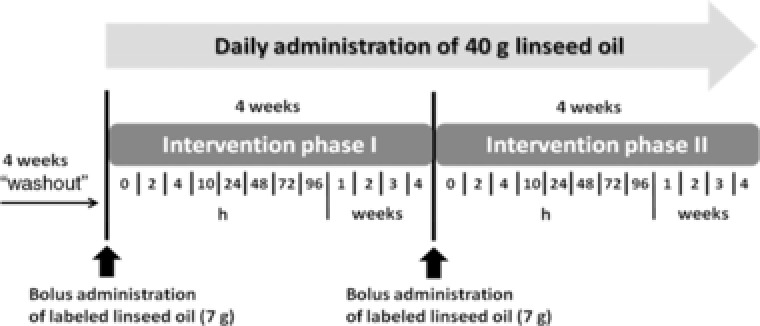
Study design. Blood was collected 0, 2, 4, 10, 24, 48, 72, and 96 h as well as 1, 2, 3, and 4 weeks after administration of the labeled LO.

Participants under free‐living conditions were required to abstain from fish and fish products as well as dietary supplements throughout the study, and were asked not to change their overall life style, e.g., level of physical activity. On day 1 of the first intervention after the washout period, subjects received an oral bolus of 7 g ^13^C‐labeled LO, which corresponds to 1.58 g ^13^C‐ALA, and a daily dose of 40 g LO, which corresponds to 19.7 g ALA after an overnight 12‐h fast (**Figure** 1). Blood was collected into EDTA tubes 0, 2, 4, 10, 24, 48, 72, 96, 168, 336, 504, and 672 h after administration of the labeled LO. At the end of the first intervention, the second phase started by administration of 7 g ^13^C‐labeled LO, followed by blood withdrawals at the same time points as in phase I. Compliance and habitual dietary energy and nutrient intakes were examined by in total 12 dietary records (one record per week), each collected over 2 days during the week and 1 day at the weekend. Since all participants were compliant to the study protocol, evaluation of nutrient intake of all of them was performed using the nutrient calculation software Prodi (Nutri‐Science GmbH, Hausach, Germany). In addition, the body weight was monitored on days when blood was withdrawn.

### 2.3 Analyses of Fatty Acids in Plasma Phospholipids, Erythrocytes, and LDL

Blood samples were centrifuged at 2000 × *g* for 15 min at 4 °C within 1 h after venipuncture. Samples were stored at –80 °C until analyses.

#### 2.3.1 Plasma Phospholipids

Plasma phospholipids were extracted according to Kolarovic and Fournier.^37^ Briefly, 10 μg of the internal standard, triheneicosanoin, and 1 mL of the antioxidant, butylhydroxy toluene (50 mg L^–1^ dissolved in methanol), were added to 0.5 mL plasma. The tubes were vortexed, and lipids were extracted four times with n‐hexane:2‐propanol (3:2, v/v) and three times with n‐hexane. The pooled extracts were dried under nitrogen and redissolved in 200 μL hexane:2‐methoxy‐2‐methylpropane:acetic acid (100:3:0.3, v/v/v). According to Agren et al.,^38^ the samples were loaded on 500 mg aminopropylsilyl SPE columns (Sigma, LC‐NH_2_, 6 mL) to isolate plasma phospholipids. Plasma phospholipids were eluted with 6 mL of methanol:chloroform:water (10:5:4, v/v/v). The collected fractions were dried under nitrogen and transmethylated by adding 1 mL of 14% boron trifluoride (in methanol) and 2 mL n‐hexane as described earlier.^39^ The methyl esters were formed after exposure of the samples to 100 °C for 1 h and extracted with hexane. After evaporating the samples to complete dryness under nitrogen, 50 μL of n‐hexane was added and subjected to analyses by means of GC fitted to flame ionization detector. The fatty acid methyl esters (FAMEs) (1 μL) were separated on an Agilent 7890A GC equipped with a capillary HP‐88 column (length: 100 m, inner diameter: 0.25 mm, film thickness: 0.2 µm). The oven temperature was set to 140 °C for 4 min and was increased by 4 °C min^–1^ to 240 °C. The injector and detector temperatures were set at 260 °C. The experiments were performed in three technical replicates. The FAMEs were identified by retention time and quantified using the internal standard triheneicosanoin.

#### 2.3.2 Erythrocytes

Erythrocytes were washed twice with 15 mL 0.9% NaCl, and 2 mL cooled water was added to lyse cells. The erythrocyte lipid extraction was carried out with 4 mL dichloromethane:methanol (2:1, v/v) after addition of 10 μg of the internal standard triheneicosanoin. The organic layer was evaporated to dryness under nitrogen and redissolved in 1 mL of NaOH in methanol (0.5 m). The transmethylation step was carried out as described above for the plasma phospholipids using 14% boron trifluoride (in methanol). The FAMEs were analyzed with an Agilent 7890A HR‐GCMS on a capillary HP‐88 column (length: 100 m, inner diameter: 0.25 mm, film thickness: 0.20 μm). The oven temperature was set to 140 °C for 4 min and was increased by 4 °C min^–1^ to 240 °C. The injector and detector temperatures were set at 250 °C. The experiments were performed in three technical replicates. Data acquisition for quantification of FAMEs was performed in the SIM mode. For quantification of the unlabeled methyl esters of ALA, EPA, DPA, and DHA, the *m/z* ions 293, 317, 345, and 343, respectively, were monitored, while the corresponding labeled esters of ALA, EPA, DPA, and DHA were analyzed by monitoring the *m/z* ions 295, 319, 347, and 345, respectively. ^13^C isotope corrections were performed by considering the intensities of all natural isotopologues. Quantification was performed by means of internal calibration using triheneicosanoin as internal standard.

#### 2.3.3 Low‐Density Lipoproteins

LDL isolation from plasma was performed by ultracentrifugation using discontinuous potassium bromide density gradient, as described previously.^30^, ^40^ Briefly, LDL was collected at a density of 1.26 g cm^–3^ using a Beckman ultracentrifuge equipped with a Beckman near vertical rotor (NVT 65 Ti‐Rotor). To separate LDL from EDTA plasma, the tubes were centrifuged for 2 h at 10 °C at 60 000 rpm. Finally, the LDL preparation was filtered through a 0.22 μm filter prior lipid extraction. Lipid extraction, lipid hydrolysis, transmethylation of fatty acids, and analyses of FAMEs were carried out as described above for erythrocytes.

### 2.4 Design of Multicompartment Model

**Figure 2:**
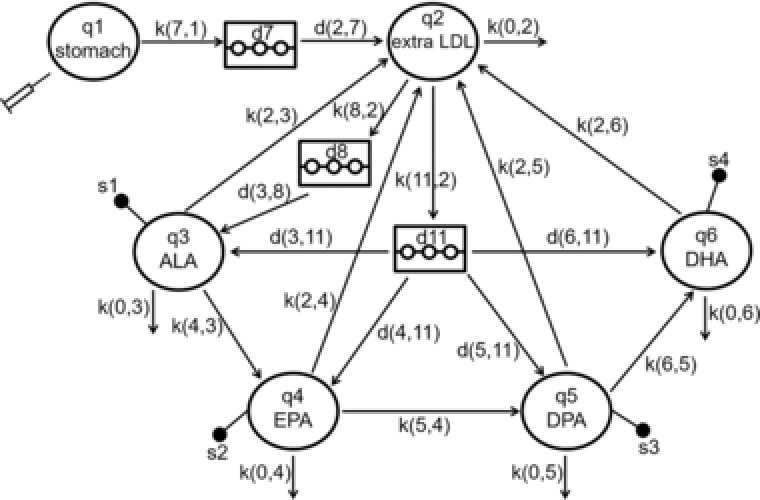
Tracer Model of ALA metabolism during the intervention phases I and II. The syringe depicts ^13^C‐ALA administration. The q1 compartment represents the dietary intake of ^13^C‐ALA, while the q2 compartment represents the ^13^C‐labeled n‐3 fatty acids in the blood. The other circles denoted q3, q4, q5, and q6 represent the ^13^C‐labeled ALA, EPA, DPA, and DHA in LDL, respectively. Two delay compartments, d8 and d11, consider the delayed transfer of the ^13^C‐n‐3 fatty acids. The delay d11 accounts for the further hepatic and extrahepatic exchange of the ^13^C‐n‐3 fatty acids. The closed circles s1–s4 represent the blood samples drawn to analyze the ^13^C‐labeled n‐3 fatty acids in LDL.

To study the temporal distribution and conversion of labeled n‐3 fatty acids (tracers) in blood, we developed a mathematical model according to Goyens et al.^24^ and Pawlosky et al.,^15^ which describes the input, output, and exchange of tracers using SAAM II software version 1.2 (SAAM Institute, Inc., Seattle, WA).^41^ The structural model is based on experimental tracer data of the current study (closed circle in **Figure** 2), a priori knowledge, and assumptions of the n‐3 fatty acid metabolism. The multicompartment model describes hepatic ^13^C‐ALA conversion to its long‐chain homologues, ^13^C‐EPA, ^13^C‐DPA, and ^13^C‐DHA in LDL, and the exchange between the labeled long‐chain n‐3 fatty acids in LDL and extra LDL compartment. The extra LDL compartment comprises all tissues capable of converting ALA besides LDL. The rate constants k(i,j), which were determined applying the current model and iterative nonlinear least‐squares of SAAM II,^41^ specify the fraction of labeled n‐3 fatty acid, which is transferred from substrate compartment j to product compartment i per hour. The rate constants k(0,j) denote an irreversible loss of the substrate from the compartment, e.g., due to oxidation of the n‐3 fatty acid. As ^13^C‐ALA was administered orally, ^13^C‐ALA reaches the extra LDL compartment after a delay of 5 h (d7), caused by the passage through the gastrointestinal tract. From the extra LDL compartment, labeled ALA can be transferred to the ALA compartment in LDL after a delay of 4 h (d8), caused by the implementation of the labeled n‐3 fatty acid into LDL. However, ^13^C‐ALA can also be converted within the extra LDL compartment to its metabolites EPA, DPA, and DHA, which can subsequently be transferred to LDL. For this pathway, a delay of 250 h (d11) resulted in the best model‐predicted fit through the experimental data. Finally, ^13^C‐labeled n‐3 fatty acids may also be irreversibly lost from the extra LDL compartment, e.g., caused by oxidation or storage of the labeled n‐3 fatty acid in adipose tissue. The hepatic conversion of ^13^C‐ALA to ^13^C‐EPA, ^13^C‐DPA, and ^13^C‐DHA is described in the LDL compartment. Loss of the labeled n‐3 fatty acids in LDL and backward transfers of the labeled n‐3 fatty acids from LDL to the extra LDL compartment were considered. Hepatic retroconversion of labeled polyunsaturated n‐3 fatty acids to its precursor n‐3 fatty acids was not included in the final model as the model predicted to the experimental data achieved best fit according to Pawlosky et al.^15^ In addition, in a recent study, it could be shown that retroconversion from DHA to EPA is a minor contributor to increased EPA levels in rat liver after a 12‐week supplementation with ALA or DHA and ALA as determined applying compound specific isotope analysis.^42^ However, in the current study, extrahepatic retroconversion of labeled n‐3 fatty acids via the extra LDL compartment was considered.

### 2.5 Quantification of ^13^C‐ALA Incorporation and Conversion

The tracer concentrations in LDL were expressed in nmol mL^–1^. To obtain the steady state mass (Mj), tracer concentrations were multiplied by the average amount of LDL of the study participants, which was 1.455 g per 5 L blood. The individual mean concentrations of each labeled n‐3 fatty acid of the study participants were used to calculate the steady state mass. The tracer flux (Ri,j) from compartment j to i (nmol h^–1^) was obtained by calculating the product of the compartmental tracer mass (Mj) and the corresponding rate constant k(i,j). The proportion of the stable isotope (Pi,j), which is transferred from compartment j to i, was determined by the ratio of k(i,j) and the total loss from compartment j. Model‐based calculations regarding ^13^C‐ALA conversion were performed for each intervention phase. Thus, total conversion rates were calculated for each intervention phase without differentiating between initial and late‐stage hepatic conversion rates. Incorporation of ALA tracer into the extra LDL compartment was derived from the model‐predicted constant d(2,7), while the incorporation of ALA isotope into ALA compartment in LDL was defined by the pathways describing the proportion of initial ^13^C‐ALA incorporation (k(8,2)) and ^13^C‐ALA incorporation after the hypothesized further hepatic and extra‐hepatic exchange (k(11,2) and d(3,11)). The amount and percentage of ALA tracer, which was converted consecutively to its metabolites, were calculated by the incorporated ^13^C‐ALA and the percentage fractional transfer (Pi,j) of ^13^C‐ALA to its metabolites.

### 2.6 Model Evaluation and Statistical Analyses

**Figure 3:**
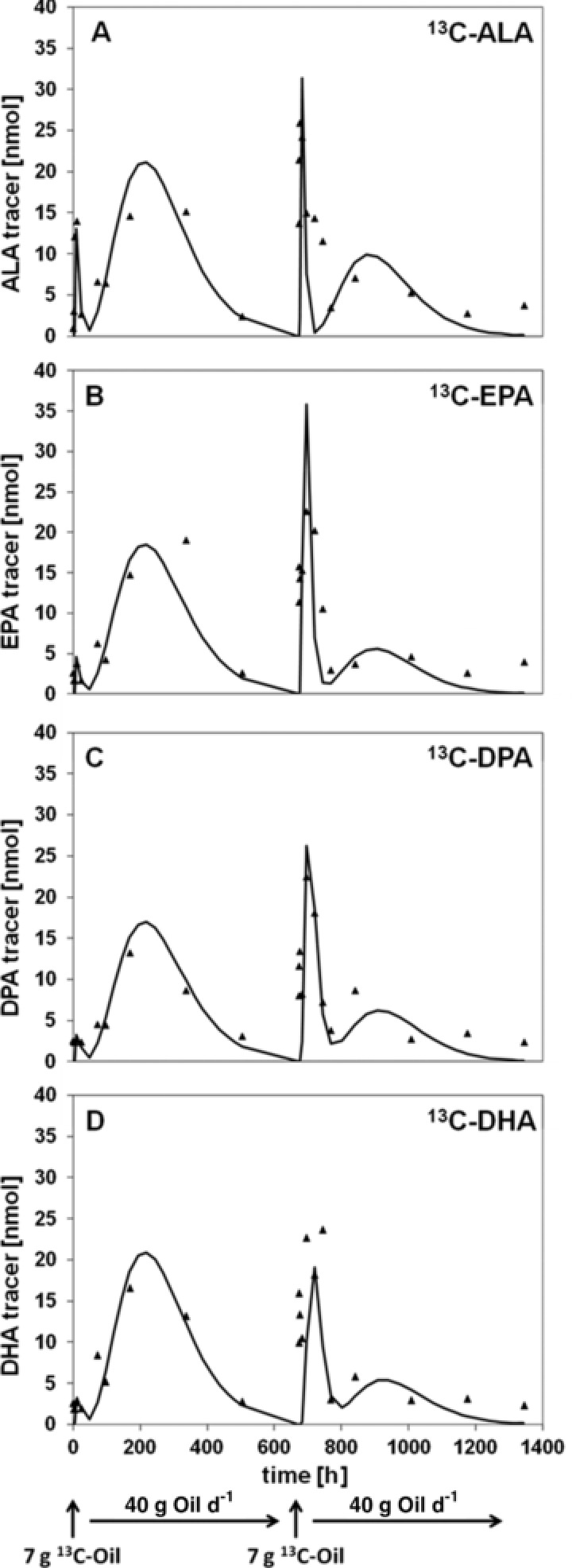
Graphical analysis of the model‐predicted fit through the experimental‐averaged data (closed triangles) during phases I and II. The amount of ALA tracer A), EPA tracer B), DPA tracer C), and DHA tracer D) was obtained applying the nonlinear least‐squares fitting.

The model was evaluated by comparing the experimental data and the model‐predicted data graphically (**Figure** 3), and numerically by calculating the standard deviations of rate constants using SAAM II (**Table** 2). Statistical analyses were carried out with Sigma Plot 11.0. All results are expressed as means ± SD or means ± SEM. Differences in energy and nutrient intakes throughout the study were analyzed by using one‐way analysis of variance (ANOVA) followed by Tukey's post hoc test. Statistical significance between before and after administration of labeled LO was determined by Kruskal–Wallis one‐way ANOVA on Ranks with Dunn's post hoc test. Differences between intervention 1 and 2 were obtained by applying the paired, two‐tailed Student's *t*‐test.

**Table 2:** Mean transfer rates (per hour) ± SD calculated for the intervention phases I and II from the mean concentrations of labeled n‐3 fatty acids observed in ten volunteers after dietary intake of ^13^C‐labeled LO using the tracer model

Rate constants	Phase I	Phase II
d (3,11)	0.01 ± 0.00	0.02 ± 0.00
d (4,11)	0.00 ± 0.01	0.00 ± 0.00
d (5,11)	0.00 ± 0.01	0.00 ± 0.00
d (6,11)	0.00 ± 0.01	0.00 ± 0.00
k (0,2)	100 ± 0.00	100 ± 0.00
k (0,3)	0.00 ± 0.00	0.00 ± 0.00
k (0,4)	0.00 ± 0.00	0.00 ± 0.00
k (0,5)	0.00 ± 0.00	0.00 ± 0.00
k (0,6)	0.00 ± 0.00	0.00 ± 0.00
k (2,3)	0.90 ± 0.00	0.90 ± 0.00
k (2,4)	0.00 ± 0.00	0.00 ± 0.00
k (2,5)	0.00 ± 0.00	0.00 ± 0.00
k (2,6)	0.95 ± 0.64	0.08 ± 0.04
k (4,3)	0.36 ± 0.24	0.18 ± 0.12
k (5,4)	0.86 ± 0.52	0.08 ± 0.00
k (6,5)	0.97 ± 0.74	0.07 ± 0.02
k (7,1)	0.20 ± 0.00	0.20 ± 0.00
k (8,2)	0.00 ± 0.00	0.01 ± 0.00
k (11,2)	0.18 ± 0.07	0.04 ± 0.02
d (2,7)	1.00 ± 0.00	1.00 ± 0.00
d (3,8)	1.00 ± 0.00	1.00 ± 0.00

## 3 Results

### 3.1 Dietary Intake and Body Weight

Dietary records were used to calculate the daily mean intake of energy, carbohydrates, proteins, fat, and n‐6 and n‐3 fatty acids. No significant changes of the dietary intake of energy, carbohydrates, and proteins were observed throughout the study (Table S1, Supporting Information). However, additional consumption of 40 g LO per day was reflected by a significant increase of total fat, and n‐6 and n‐3 fatty acids intake. Mean body weight of the study subjects was 79.7 ± 10.9 kg at the beginning and 80.7 ± 11.0 kg at the end of the intervention (*p* > 0.05).

### 3.2 n‐3 PUFA Composition of Plasma Phospholipids, Erythrocytes, and LDL

**Figure 4:**
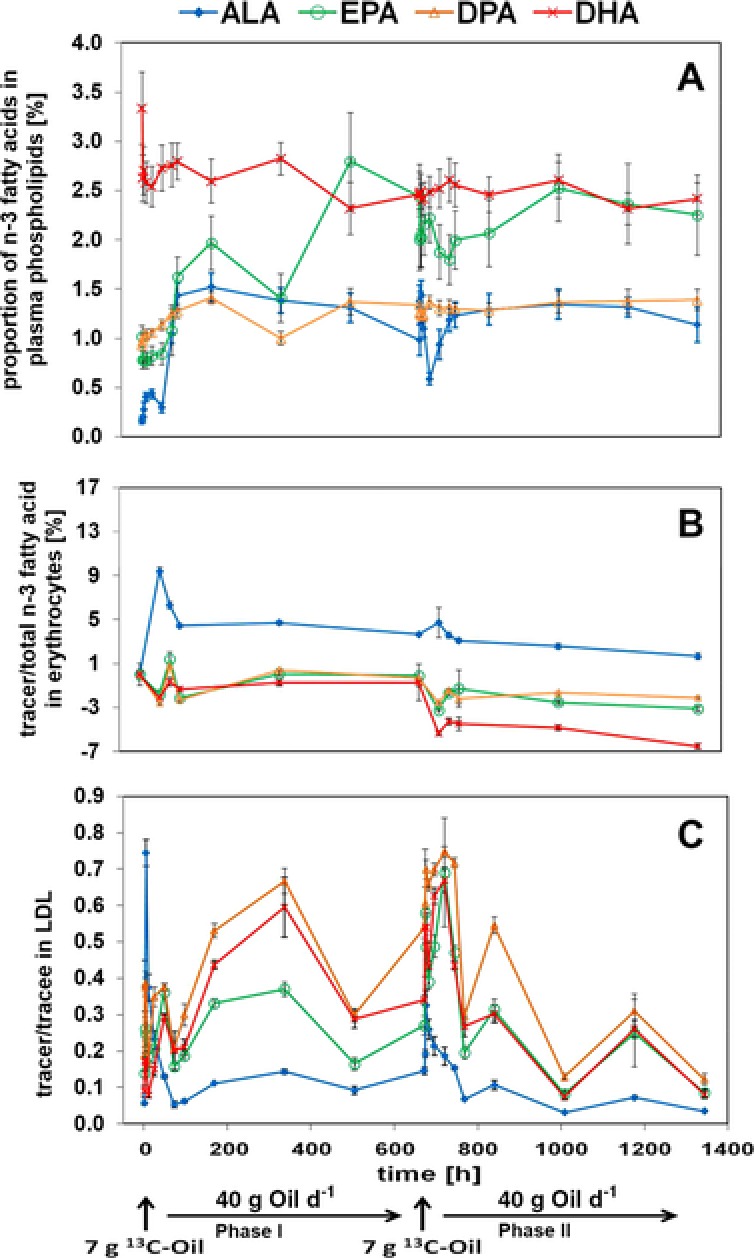
Time‐dependent courses of proportion of n‐3 fatty acids in plasma phospholipids A) and tracer/total n‐3 fatty acid in erythrocytes B) and tracer/trace in LDL C) after oral administration of 7 g ^13^C‐labeled LO at the beginning of intervention phases I and II. Blood was drawn in each intervention phase 0, 2, 4, 10, 24, 48, 72, 96, 168, 336, 504, and 672 h after administration of the labeled LO. The corresponding n‐3 fatty acids were analyzed in the respective compartment.

Prior to quantification of ^13^C‐ALA incorporation and conversion, the compartment that most reliably described ALA incorporation and conversion among plasma phospholipids, erythrocytes, and LDL was identified by analyzing n‐3 fatty acid composition during intervention phases I and II (**Figure** 4).

In plasma phospholipids, ALA and EPA proportion of total fatty acids was significantly enhanced up to 1.52 ± 0.14% (ANOVA, *p* < 0.05) at 168 h and 2.80 ± 0.49% (ANOVA, *p* < 0.05) at 504 h after LO intake, whereas the proportion of DPA remained unchanged throughout the study, except for the time point 168 h post load. Similarly, no significant changes were obtained for DHA levels throughout the study (Figure 4A).

In erythrocytes, a significant rise of the ratio of ALA tracer‐(ALA isotope)‐to‐total ALA (unlabeled and labeled ALA) by 9.38 ± 0.38% (ANOVA, *p* < 0.05) appeared 48 h after administration of the labeled LO (Figure 4B). No significant increase of labeled EPA, DPA, and DHA could be detected after administration of the labeled LO. Interestingly, a decrease of the tracer‐to‐total n‐3 fatty acid values of EPA, DPA, and DHA was monitored from the start to the end of the study by 3.12 ± 0.20%, 2.10 ± 0.12%, and 6.52 ± 0.25%, respectively (ANOVA, *p* < 0.05). After long‐term administration of unlabeled LO during intervention phase I, ^13^C‐ALA incorporation following bolus administration of ^13^C‐labeled LO was limited during intervention phase II, as evident by a 49.9 ± 14.5% (ANOVA, *p* < 0.05) less pronounced increase of n‐3 fatty acid tracer‐to‐total n‐3 fatty acid ratio after dietary intake of labeled LO at the beginning of intervention phase II compared to the rise after bolus administration in phase I.

In LDL, an up to 13.2 ± 0.65 fold, 5.00 ± 1.08 fold, 3.95 ± 0.08 fold, and 7.50 ± 0.38 fold (ANOVA, *p* < 0.05) increase of ALA, EPA, DPA, and DHA tracer‐to‐tracee (unlabeled n‐3 fatty acid) ratio, respectively, was analyzed after administration of the ^13^C‐labeled study oil (Figure 4C). A limited incorporation of 56.4 ± 5.63% (ANOVA, *p* < 0.05) of ALA tracer‐to‐tracee, which was administered in phase I, was demonstrated in LDL after preconditioning with ALA in phase II by setting the initial ALA tracer‐to‐tracee peak in phase I (4 h) to 100% and calculating the percentage of the ALA tracer‐to‐tracee peak 4 h after the administration of the ^13^C‐labeled study oil in phase II (676 h). Since the most pronounced increase of DHA levels after administration of ^13^C‐labeled LO was achieved in the LDL compartment, as compared to plasma phospholipids and erythrocytes, quantification of ^13^C‐ALA incorporation and conversion was performed in LDL by means of compartmental modeling.

### 3.3 Effect of ALA Preconditioning on ALA Incorporation and Conversion Quantified by Compartmental Modeling in LDL

Figure 2 represents the compartmental model used to quantify ^13^C‐ALA incorporation and conversion to its long‐chain homologues as reflected in LDL after dietary intervention with ^13^C‐labeled LO. Concentration–time curves for ^13^C‐labeled n‐3 fatty acids were obtained by applying the model‐predicted fit to the experimental n‐3 fatty acid data (Figure 3). The model‐predicted fit represents the optimized agreement between the experimental and the predicted data. By solving the mathematical model using the mean values of labeled n‐3 fatty acids after consumption of the labeled study oil, the rate constants and their model‐based variation were calculated for phases I and II (Table 2). As presented in Table S2, Supporting Information, the individual model‐determined transfer rates were calculated for intervention phases I and II to examine the interindividual variations. The transfer rate coefficients, which describe the fraction of labeled n‐3 fatty acids that were transferred from ALA to EPA (k(4,3)), EPA to DPA (k(5,4)), and DPA to DHA (k(6,5)) compartment per hour as reflected in LDL, were significantly reduced by 59.5 ± 4.76%, 90.7 ± 0.00%, and 91.4 ± 1.08%, respectively, after preconditioning with 40 g unlabeled LO per day for 4 weeks compared to intervention phase I. As a consequence, the mean transfer rate of labeled DHA to the extra LDL compartment (k(2,6)) was also decreased by 88.9 ± 1.11% in phase II. Similarly to ^13^C‐ALA conversion as reflected in LDL, the transfer rates of ^13^C‐EPA (d(4,11)), ^13^C‐DPA (d(5,11)), and ^13^C‐DHA (d(6,11)) from the blood to the LDL compartment were significantly lowered by 100 ± 0.01%, 100 ± 0.01%, and 80.0 ± 9.73% in the intervention phase II compared to intervention phase I. However, the rate constants of the initial ^13^C‐ALA incorporation (k(8,2)) and the ^13^C‐ALA incorporation (d(3,11)) were enhanced by 143 ± 18.1% and 60.9 ± 1.10% in the intervention phase II. Notably, the transfer rate of the labeled n‐3 fatty acids in the intervention phase II (k(11,2)) was decreased by 75.0 ± 6.25%, leading to more than 45.5 ± 7.58% reduction of total ^13^C‐ALA incorporation after long‐term administration of unlabeled 19.7 g ALA per day.

Comparable results were obtained regarding the flow rate of labeled fatty acids (**Table** 3). The flow of ^13^C‐ALA to ^13^C‐EPA (R(4,3)), ^13^C‐EPA to ^13^C‐DPA (R(5,4)), and ^13^C‐DPA to ^13^C‐DHA (R(6,5)) was substantially lowered by 36.1 ± 5.27%, 85.2 ± 1.79% and 89.1 ± 1.02% after a 2‐month intake of unlabeled LO. The reduced mass of ^13^C‐ALA conversion also caused a diminution of the flow of ^13^C‐DHA to the extra LDL compartment (R(2,6)) by 85.0 ± 2.97%. Likewise for the transfer rate, the flow of the initial ^13^C‐ALA incorporation (R(8,2)) was enhanced by 117 ± 14.8% after the interventions during phase I. However, the backward flow of ^13^C‐ALA from ALA to the extra LDL compartment (R(2,3)) was also enhanced by 24.0 ± 1.98% in phase II.

**Table 3:** Compartmental masses ± SEM and tracer fluxes ± SEM during the intervention phases I and II

Compartmental masses and tracer fluxes	Phase I	Phase II
Steady state mass for tracer [nmol]		
M_3_ (ALA)	13.9 ± 1.37^a^	17.2 ± 1.42^b^
M_4_ (EPA)	9.78 ± 0.80^a^	15.5 ± 1.90^b^
M_5_ (DPA)	9.16 ± 0.38^a^	13.4 ± 1.22^b^
M_6_ (DHA)	9.58 ± 0.77^a^	16.4 ± 3.22^b^
Tracer fluxes [nmol h^–1^]		
R_(7,1)_ = R_(2,7)_	11.2 × 10^5a^	11.2 × 10^5a^
R_(8,2)_ = R_(3,8)_	23.7 × 10^3^ ± 2.43 × 10^3a^	51.5 × 10^3^ ± 6.51 × 10^3b^
R_(4,3)_	4.93 ± 0.49^a^	3.15 ± 0.26^b^
R_(5,4)_	8.38 ± 0.69^a^	1.24 ± 0.15^b^
R_(6,5)_	8.84 ± 0.37^a^	0.96 ± 0.09^b^
R_(2,3)_	12.5 ± 1.23^a^	15.5 ± 1.28^b^
R_(2,4)_	0.00 ± 0.00^a^	0.00 ± 0.00^a^
R_(2,5)_	0.00 ± 0.00^a^	0.00 ± 0.00^a^
R_(2,6)_	9.09 ± 0.73^a^	1.36 ± 0.27^b^
R_(0,3)_	0.00 ± 0.00^a^	0.00 ± 0.00^a^
R_(0,4)_	0.00 ± 0.00^a^	0.00 ± 0.00^a^
R_(0,5)_	0.00 ± 0.00^a^	0.00 ± 0.00^a^
R_(0,6)_	0.00 ± 0.00^a^	0.0 ± 0.00^a^

Statistical significant differences between the two phases were analyzed by a two‐sided, paired *t*‐test, and are indicated with different superscript letters (*p* < 0.01).

A 2‐month intake of the unlabeled, ALA‐rich LO not only affected the transfer rates and the fluxes, but also induced a marked augmentation of the steady state mass by 23.7 ± 1.96%, 58.5 ± 7.17%, 46.3 ± 4.22%, and 71.2 ± 14.0% for the ALA (M3), EPA (M4), DPA (M5), and DHA (M6) compartment compared to intervention phase I (Table 3).

The mean percentage fractional transfer of ^13^C‐ALA conversion to ^13^C‐EPA (P(4,3)) yielded 30.6 ± 2.82% based on individual data, thereby considering the interindividual variations, in phase I. This high fractional transfer showed a pronounced reduction by 48.0 ± 5.59% after preconditioning of ten subjects with 40 g unlabeled LO per day for 4 weeks (**Table** 4). The ^13^C‐ALA incorporation (P(8,2)) after bolus administration was enhanced by 143 ± 18.1% after the first 4 weeks of intervention. However, the mean percentage fractional transfer of ^13^C‐ALA from LDL to the extra LDL compartment (P(2,3)) was also slightly elevated by 21.2 ± 0.43% after administration of the second bolus dose. Incorporation of ^13^C‐ALA after administration of the second bolus dose (P(3,11)) was increased by 59.9 ± 1.35% in phase II, while the secondary incorporation of ^13^C‐EPA, ^13^C‐DPA, and ^13^C‐DHA was markedly decreased by 100 ± 0.001%, 100 ± 0.001%, and 82.5 ± 9.12%, respectively. Noteworthy, the ^13^C‐labeled n‐3 fatty acid fractional transfer from the blood to the LDL compartment (P(11,2)) was negatively affected by a two‐2 treatment with high amounts of LO, resulting in a 75.0 ± 6.25% reduction.

**Table 4:** Mean percentage fractional transfers P (%) ± SEM during intervention phases I and II

	Phase I		Phase II	
	Based on averaged data	Based on individual data	Based on averaged data	Based on individual data
P_(8,2)_	0.00	0.00 ± 0.00^a^	0.01	0.01 ± 0.00^b^
P_(11,2)_	0.18	0.16 ± 0.01^a^	0.04	0.04 ± 0.01^b^
P_(3,11)_	68.3	69.4 ± 4.26^a^	99.9	111 ± 2.50^b^
P_(4,11)_	21.4	16.6 ± 4.07^a^	0.00	0.00 ± 0.00^b^
P_(5,11)_	1.31	8.61 ± 2.80^a^	0.00	0.00 ± 0.00^b^
P_(6,11)_	8.96	5.37 ± 1.88^a^	0.09	0.94 ± 0.49^b^
P_(4,3)_	28.3	30.6 ± 2.82^a^	16.9	15.9 ± 1.71^b^
P_(5,4)_	100	100 ± 0.00^a^	100	100 ± 0.00^a^
P_(6,5)_	100	100 ± 0.00^a^	100	100 ± 0.00^a^
P_(2,6)_	100	100 ± 0.00^a^	100	100 ± 0.00^a^
P_(2,5)_	0.00	0.00 ± 0.00^a^	0.00	0.00 ± 0.00^a^
P_(2,4)_	0.00	0.00 ± 0.00^a^	0.00	0.00 ± 0.00^a^
P_(2,3)_	71.7	69.4 ± 2.82^a^	83.1	84.1 ± 1.71^b^
P_(0,3)_	0.00	0.00 ± 0.00^a^	0.00	0.00 ± 0.00^a^
P_(0,4)_	0.00	0.00 ± 0.00^a^	0.00	0.00 ± 0.00^a^
P_(0,5)_	0.00	0.00 ± 0.00^a^	0.00	0.00 ± 0.00^a^
P_(0,6)_	0.00	0.00 ± 0.00^a^	0.00	0.00 ± 0.00^a^

Statistical significant differences between the two phases were analyzed by a two‐sided, paired *t*‐test and are indicated with different superscript letters (*p* < 0.05).

According to the model‐based mathematical solution, incorporation of the ALA tracer into the extra LDL compartment was assumed as 100% (**Table** 5), thereby confirming other studies.^17^ The total incorporation of ingested ^13^C‐ALA into ALA compartment in LDL solely yielded 6.19 ± 0.61 μmol in phase I. This low incorporation was even further diminished by 45.5 ± 7.58% in phase II. The incorporated ^13^C‐ALA was converted to its long‐chain homologues as reflected in LDL compartment, yielding 1.95 ± 0.37 μmol of the metabolites in phase I. Preconditioning with the unlabeled ALA‐rich LO for 4 weeks resulted in a significant decrease of the amount of dietary ^13^C‐ALA converted to its metabolites by 72.5 ± 3.30%.

**Table 5:** Incorporation of the ^13^C‐labeled ALA tracer into the extra LDL and LDL‐ALA compartment, and conversion of the ALA tracer during both intervention phases

	Intervention phase I	Intervention phase II
Dietary intake of ALA tracer [mmol]	5.62	5.62
Incorporation of ALA tracer into extra LDL compartment	100% 5.62 mmol	100% 5.62 mmol

	Based on averaged data	Based on individual data	Based on averaged data	Based on individual data
Incorporation of ALA tracer into ALA compartment in LDL	0.13% 7.06 μmol	0.11 ± 0.01%^a^ 6.19 ± 0.61 μmol^a^	0.06% 3.26 μmol	0.06 ± 0.01%^b^ 3.24 ± 0.31 μmol^b^
Conversion of ALA into EPA tracer	0.04% 2.00 μmol	0.03 ± 0.01%^a^ 1.95 ± 0.37 μmol^a^	0.01% 0.55 μmol	0.01 ± 0.00%^b^ 0.50 ± 0.06 μmol^b^
Conversion of ALA into DPA tracer	0.04% 2.00 μmol	0.03 ± 0.01%^a^ 1.95 ± 0.37 μmol^a^	0.01% 0.55 μmol	0.01 ± 0.00%^b^ 0.50 ± 0.06 μmol^b^
Conversion of ALA into DHA tracer	0.04% 2.00 μmol	0.03 ± 0.01%^a^ 1.95 ± 0.37 μmol^a^	0.01% 0.55 μmol	0.01 ± 0.00%^b^ 0.50 ± 0.06 μmol^b^

Statistical significant differences between the two phases were analyzed by a two‐sided, paired *t*‐test and are indicated with different superscript letters (*p* < 0.01).

## 4 Discussion

The aim of the current study was to investigate the effect of a 1‐ and 2‐month intake of an ALA‐rich LO on ALA incorporation and conversion in healthy subjects. In general, ALA conversion can be increased by administration of high amounts of ALA, in particular when ingested with a diet low in n‐6/n‐3 and high in dietary fat.^14^, ^25^, ^43^ However, evidence for the effects of long‐term intake of ALA‐rich vegetable oils with a low n‐6/n‐3 fatty acid ratio on ALA conversion rates is scarce. LO was chosen as an ALA‐rich source due to its n‐6/n‐3 fatty acid ratio of approximately 1:3.^8^ In previous studies, labeled free fatty acids^12^, ^13^, ^24^ and labeled ethyl esters^5^, ^15^, ^16^ were used to calculate the conversion of ALA to its longer‐chain polyunsaturated fatty acids. However, despite the known differences in the bioavailability of fatty acids, in particular when ingested as free fatty acids, ethyl esters, or triglycerides,^11^ studies aiming at the quantification of n‐3 fatty acid incorporation and metabolism after administration of isotopically labeled vegetable oils are missing. In the current study, isotopically labeled LO was used to quantify ^13^C‐ALA incorporation and conversion rate.

### 4.1 Identification of Compartment to Study Incorporation and Conversion of ALA

Prior quantification of ^13^C‐ALA incorporation and conversion after consumption of ^13^C‐labeled and nonlabeled LOs, the present study investigated whether increase of ALA conversion would be best reflected in plasma phospholipids, erythrocytes, or LDL. Although most of the studies focus on the short‐term effects of ALA supplementation on its incorporation and conversion within 96 h post‐dose, the current study elucidated the long‐term effects within 1344 h (8 weeks). LDL proved to be the most sensitive compartment to study hepatic ALA conversion. So far, LDL has not been considered as a compartment for studying ALA conversion in the literature, although it might best reflect hepatic conversion of ALA due to its site of production. LDL derives from VLDL, which is exported from the liver. Comparably higher amounts of LDL than VLDL were expected to allow more reliable quantification of ALA incorporation and conversion. Goyens et al.^24^ chose plasma phospholipids to quantify ALA conversion by compartmental modeling in 29 healthy volunteers who consumed a diet with an ALA:LA ratio of 1:19 for 28 days. Results from Goyens et al.^24^ demonstrated that only 0.073 ± 0.04% of the incorporated ALA was converted to DHA. As shown in Figure 1A, we could not observe any significant changes for the DHA levels in plasma phospholipids throughout the study. This slight discrepancy cannot be explained by the difference in the supply with ^13^C‐ALA. Although Goyens et al.^24^ provided ^13^C‐ALA as free fatty acid, the present tracer study used ^13^C‐labeled LO, of which the bioavailability is higher than that of free fatty acids, as suggested by Beckermann et al.^11^ As in the current study high amounts of ALA (19.7 g) were administered while Goyens et al.^24^ only provided approximately one‐tenth lower amounts of labeled ALA, high amounts of ALA might limit the conversion efficiency of ALA. This hypothesis is corroborated by Gregory et al.,^44^ who showed that ALA at high amounts compete with tetracosapentaenoic acid, a precursor of DHA, for fatty acid desaturase 2.

Sun et al.^45^ suggested the fatty acid content of erythrocytes being the preferred biomarker for reflecting the long‐term fatty acid intake, as, first, erythrocytes were less sensitive to recent changes in fatty acid intake, and, second, a higher correlation between fatty acid intake and DHA content of erythrocytes (*r* = 0.56) compared to plasma (*r* = 0.48) was identified in 306 U.S. women aged 43–69 years.^45^ Katan et al.^46^ also proposed erythrocytes as biomarkers to reflect dietary intake of n‐3 fatty acids. They performed a human intervention trial with 58 men, who were asked to consume 0, 3, 6, or 9 g fish oil per day for 12 months, to quantify the incorporation of n‐3 fatty acids with respect to their intake. Their findings suggest that the EPA content in erythrocytes reflect the fish oil intake over the past 1–2 months most reliably. In the current study, an initial rise of the ALA tracer‐to‐tracee levels by 9.38 ± 0.38% was obtained 48 h after dietary intake of the labeled LO, whereas no significant increase of the corresponding EPA, DPA, and DHA metabolites could be observed, which is in agreement with results of one of our own human intervention studies published recently.^47^ According to the above mentioned studies,^45^, ^46^, ^47^ the fatty acid content of the erythrocytes does reflect the intake of fatty acids, although erythrocytes may not be the most sensitive compartment for analyzing dietary ALA conversion. In our study, it was demonstrated that the tracer‐to‐total n‐3 fatty acid values of EPA, DPA, and DHA were markedly reduced by 3.12 ± 0.20%, 2.10 ± 0.12%, and 6.52 ± 0.25% in erythrocytes, respectively, suggesting that EPA, DPA, and DHA were replaced by ALA in response to long‐term treatment with high dose of ALA.

### 4.2 Effects of Administration of ALA‐rich LO on Incorporation of ^13^C‐ALA in LDL

In the current study, ^13^C‐ALA incorporation and conversion into its long‐chain polyunsaturates was determined by applying multicompartment modeling after dietary intake of the ^13^C‐labeled LO. A structural model was designed based on physiological a priori knowledge. This model was validated with respect to the variation of the model‐predicted data from the experimental data. A model was developed for the quantification of ALA incorporation and conversion as reflected in LDL. In addition, the model considers not only the initial rise of ^13^C‐ALA after administration of ^13^C‐labeled LO, but also the rise of ^13^C‐ALA observed after the hypothesized further hepatic and extrahepatic exchange, which is not taken into account in other studies focusing on ALA incorporation.

Long‐term administration of the unlabeled ALA‐rich LO for 4 weeks resulted in a reduced total incorporation of the administered 1.58 g ^13^C‐ALA in LDL compared to phase I (no preconditioning). Recently, Harnack et al.^20^ revealed that incorporation of ^13^C‐ALA into HepG2 cells treated with varying ratios of ^13^C‐linoleic acid and ^13^C‐ALA for 24 h is saturable, yielding the highest recovery of 84% with a ratio of ^13^C‐linoleic acid and ^13^C‐ALA of 4:1. A saturation of ^13^C‐ALA incorporation might also be the reason for the limited total incorporation of 0.11 ± 0.01% in the current human intervention trial, after administration of 1.58 g of ^13^C‐ALA. Goyens et al.^24^ provided 10 mg of ^13^C‐ALA as free fatty acid twice per day for 8 days after an initial bolus of 30 mg of the same ^13^C‐ALA preparation and calculated an incorporation of ALA into plasma phospholipids of almost 7%. In the present study, the volunteers received a bolus of 1.58 g ^13^C‐ALA and a daily dose of 19.7 g ALA. Despite this high load of ALA, the incorporation rate in our study (0.11%) was lower than in the study performed by Goyens et al.^24^ (7%). The low total incorporation of ^13^C‐ALA into LDL during the phase I of the current study was further reduced by 45.5 ± 7.58% after long‐term dietary intake of the unlabeled ALA‐rich LO. This reduction of total ^13^C‐ALA incorporation after preconditioning with the unlabeled ALA‐rich LO might be explained by a higher rate of ALA metabolism. However, we observed a slower turnover rate in intervention phase II. Thus, we hypothesize that high amounts of dietary ALA might shift ALA metabolism toward ß‐oxidation. Excess of ALA may trigger the ß‐oxidation rate of ALA and ^13^C‐ALA in the liver. Burdge et al.^12^ reported that the intake of 9.55 ± 0.83 mg ALA per day in an 8 week intervention did not alter partitioning of ALA toward ß‐oxidation compared to preintervention conditions with an average habitual intake of 1.67 ± 0.77 mg ALA per day. Moreover, the authors demonstrated that ALA conversion was downregulated by increased product (EPA + DHA) availability. Although the results presented by Burdge et al.^12^ indicate that neither ALA conversion nor ß‐oxidation of ALA might be upregulated by increased ALA consumption alone, a dose‐dependent effect cannot be excluded. In the present study, a total amount of 19.7 g ALA was ingested by means of the unlabeled LO (40 g), which might have stimulated ALA conversion, ß‐oxidation, and, possibly, eicosanoid synthesis. This hypothesis is supported by results from McCloy et al.^22^ who demonstrated that more than 60% of a bolus dose of 47 mg ^13^C‐ALA were subjected to ß‐oxidation, explaining the low incorporation rate prior and after preconditioning with ALA in the current study.

The compartment model presented in this study not only considers total ALA incorporation, but also discriminates between ALA incorporation after administration of ^13^C‐labeled LO without and with a 4‐week preconditioning phase with an ALA‐rich LO. Preconditioning with the ALA‐rich LO‐enhanced ^13^C‐ALA incorporation (P(8,2)) by 143 ± 18.1%, whereas the ^13^C‐labeled n‐3 fatty acid fractional transfer after the hypothesized further hepatic and extrahepatic exchange from the blood to the LDL compartment (P(11,2)) decreased after long‐term treatment with high amounts of LO, resulting in a 75.0 ± 6.25% reduction compared to short‐term treatment with ALA‐rich LO in intervention phase I. Here, we hypothesize that the reduction of the labeled n‐3 fatty acid incorporation after further hepatic and extrahepatic exchange might also be due to enhanced ß‐oxidation. However, no breath samples have been drawn during the intervention phases to examine the role of ß‐oxidation, which is a limitation of the present study.

### 4.3 Effect of Administration of ALA‐rich LO on ALA Conversion

After studying the effect of a 1‐ and 2‐month intake of the ALA‐rich LO on ^13^C‐ALA incorporation, ^13^C‐ALA conversion to its longer‐chain polyunsaturates was investigated. Total fractional ^13^C‐ALA conversion to ^13^C‐EPA was calculated to be 30.6 ± 2.82%, which is within the range described in the literature,^13^, ^23^, ^24^ whereas high‐dose administration of 17 g ALA per day to 38 hyperlipidemic men for 12 weeks yielded a fractional conversion of ALA to EPA of only 0.29% in erythrocyte phospholipids,^13^ remarkably higher fractional conversion rates from ALA to EPA of 21% were analyzed in plasma lipids after administration of 0.7 g of ^13^C‐ALA as free fatty acid to healthy volunteers by Burdge and Wootton.^23^ The highest fractional ALA conversion to EPA of 99.81 ± 0.29% has been reported by Goyens et al.^24^ for plasma phospholipids after bolus administration of 30 mg ^13^C‐ALA to healthy subjects. This broad range of values observed for the fractional conversion of ALA to EPA might be explained by the differences in the intervention time, the amount of ALA administered, and the compartment analyzed (erythrocytes vs plasma vs plasma phospholipids). One of the more widely accepted hypotheses is that long‐term high intake of ALA might inhibit its conversion to EPA.^13^ This hypothesis could be confirmed by the results obtained from the current study. Preconditioning with 19.7 g ALA from 40 g LO per day for 4 weeks resulted in a reduced total tracer flux and total fractional conversion of ^13^C‐ALA to ^13^C‐EPA by 36.1 ± 5.27% and 48.0 ± 5.59%, respectively, compared to the total tracer flux and the total fractional conversion of ^13^C‐ALA to ^13^C‐EPA in the intervention phase I. Regarding the maximum of the initial peak after 676 h in the intervention phase II (Figure 3), almost 90% of ^13^C‐ALA was converted to ^13^C‐EPA, which is approximately 3 times more than the percentage calculated for intervention phase I. Vermunt et al.^35^ also demonstrated that high dose administration of 8.3 g ALA per day for 6 weeks followed by a bolus of 45 mg ^13^C‐ALA led to reduced contents of 0.04 ± 0.01 mg ^13^C‐EPA in plasma total lipids compared to a diet rich in oleic acid (0.12 ± 0.03 mg ^13^C‐EPA).

Regarding the hepatic desaturation and elongation of ^13^C‐EPA to ^13^C‐DPA and ^13^C‐DHA as reflected in LDL particles, the mathematical model applied in the present study revealed that 100% of ^13^C‐EPA formed is transferred to ^13^C‐DPA and ^13^C‐DHA, in either of the intervention phases. Pawlosky et al.^15^ calculated the fractional conversion rates for ALA to EPA, EPA to DPA, and DPA to DHA in the plasma with 0.2%, 65%, and 37%, respectively, after asking eight healthy subjects on a controlled beef‐based diet to consume a bolus of 1 g of d5‐labeled ALA as ethyl ester. In contrast, Goyens et al.^24^ achieved fractional conversion rates in plasma phospholipids for EPA to DPA of merely 1.05 ± 0.52%, whereas 99.8 ± 0.29% of ALA were converted to EPA, although the first reaction of ALA to EPA is the rate‐limiting step of the pathway. The fractional conversion of DPA to DHA also yielded 100%.

On the other hand, previous studies reported rather low conversion of ALA to DHA in plasma and erythrocytes, ranging from 0 to 3.8%,^15^, ^24^, ^27^, ^28^, ^29^, ^30^, ^31^, ^48^ the present study shows that hepatic conversion of ^13^C‐ALA to ^13^C‐DHA, as reflected by the LDL compartment, yielded a total percentage fractional transfer of 30.6 ± 2.82%. Here, we conclude the extent of total fractional ALA conversion to DHA being specific for the compartment analyzed, with highest fractional conversion rates of ALA to DHA being reflected in LDL. Preconditioning with 19.7 g ALA by means of 40 g LO per day for 4 weeks induced a marked decrease of the total mean percentage fractional transfer of ^13^C‐ALA to ^13^C‐DHA by 48.0 ± 5.59%. This decrease in ^13^C‐DHA concentration after long‐term consumption of high amounts of ALA might be attributed to competitive substrate inhibition.^49^ It is well known that Δ6‐desaturase catalyzes the conversion of ALA to stearidonic acid, but also the desaturation of tetracosapentaenoic to tetracosahexaenoic acid.^44^ Both reactions yield DHA. The competition between ALA and tetracosapentaenoic acid for Δ6‐desaturase activity might explain the reduced DHA formation under conditions of ALA excess. The saturation of the elongation of DPA to tetracosapentaenoic acid might also explain the reduced fractional conversion rate to DHA after high dose administration of ALA.^44^ However, saturation of this elongation reaction would lead to accumulation of DPA, which is not the case in the current study, favoring the explanation of competition for the Δ6‐desaturase. It needs to be noted that including a group who would have been treated with lower ALA doses might have clarified the role of competitive substrate inhibition.

## 5 Concluding Remarks

The present study investigated the effect of preconditioning with an ALA‐rich LO on ^13^C‐ALA incorporation and conversion as reflected by changes in ALA, EPA, DPA, and DHA in plasma phospholipids, erythrocytes, and LDL. By applying an adapted compartment model and isotopically labeled LO, it could be shown that quantification of ^13^C‐ALA incorporation and conversion can be most reliably performed in LDL. A fractional ^13^C‐ALA conversion to ^13^C‐DHA of approximately 30% is reported for LDL. However, ^13^C‐ALA incorporation and conversion was substantially lowered by preconditioning with an ALA‐rich LO for 4 weeks, suggesting a shift from ^13^C‐ALA conversion to ß‐oxidation.

## Conflict of Interest

The authors declare no conflict of interest.

## Supporting information


**Table S1**: Mean daily intake ± SD of energy, carbohydrates, proteins, total fat, as well as n‐3 and n‐6 fatty acids of 10 healthy volunteers prior and during the intervention study at the end of each phase.
**Table S2**: Mean transfer rates (h^‐1^) ± SEM calculated for the intervention phases I and II from the individual concentrations of labeled n‐3 fatty acids observed in 10 individual volunteers after dietary administration of ^13^C‐labeled linseed oil using the tracer model.
**Figure S1**. Proportion of ^13^C‐labeling of ALA in flax seeds harvested from flax plants exposed to ^13^C‐CO_2_. The x‐axis represents the number of ^13^C‐labeled atoms in the ALA molecule.
