# Supplementary material for: Effect of 1‐ and 2‐Month High‐Dose Alpha‐Linolenic Acid Treatment on 13C‐Labeled Alpha‐Linolenic Acid Incorporation and Conversion in Healthy Subjects
Source: Mol Nutr Food Res. 2018 Sep 19;62(20):1800271. doi: 10.1002/mnfr.201800271 (PMC6646899; doi:10.1002/mnfr.201800271)
Supplement: Supplementary file 1 — Table S1: Mean daily intake ± SD of energy, carbohydrates, proteins, total fat, as well as n‐3 and n‐6 fatty acids of 10 healthy volunteers prior and during the intervention study at the end of each phase. Table S2: Mean transfer rates (h‐1) ± SEM calculated for the intervention phases I and II from the individual concentrations of labeled n‐3 fatty acids observed in 10 individual volunteers after dietary administration of 13C‐labeled linseed oil using the tracer model. Figure S1. Proportion of 13C‐labeling of ALA in flax seeds harvested from flax plants exposed to 13C‐CO2. The x‐axis represents the number of 13C‐labeled atoms in the ALA molecule. [file MNFR-62-na-s001.docx]

Supporting information

Effect of one and two months high dose alpha-linolenic acid treatment on ^13^C-labeled alpha-linolenic acid incorporation and conversion in healthy subjects

Marc Pignitter^1^, Michael Lindenmeier^2^, Gaby Andersen^2^, Cornelia Herrfurth^3^, Christopher Beermann^4^, Joachim J. Schmitt^4^, Ivo Feussner^3^, Martin Fulda^3^ and Veronika Somoza^1,2,^*

^1^ Department of Physiological Chemistry, Faculty of Chemistry, University of Vienna, Austria

^2^ German Research Center of Food Chemistry, Freising, Germany

^3^ Department for Plant Biochemistry, Albrecht-von-Haller-Institute for Plant Sciences, Georg-August-University, Goettingen, Germany

^4^ Department of Food Technology, University of Applied Sciences, Fulda, Germany

* Corresponding author: Prof. Dr. Veronika Somoza, Department of Physiological Chemistry, University of Vienna, 1090 Vienna, Austria

email: [veronika.somoza@univie.ac.at](mailto:veronika.somoza@univie.ac.at)

fax: +43-1-4277-9706

**Supplementary tables**

**Table S1:** Mean daily intake ± SD of energy, carbohydrates, proteins, total fat, as well as n-3 and n-6 fatty acids of 10 healthy volunteers prior and during the intervention study at the end of each phase.

|  | **Energy [kcal/d]** | **Carbo-hydrates [g/d]** | **Proteins [g/d]** | **Total fat [g/d]** | **n-3 fatty acids** | **n-6 fatty acids** |
| --- | --- | --- | --- | --- | --- | --- |
|  |  |  |  |  |  |  |
| **Run-in**  **Phase** | **2266 ± 375^a^** | **274 ± 61.0^a^** | **80.0 ± 20.0^a^** | **80.0 ± 28.0^a^** | **1.30 ± 0.70^a^** | **8.30 ± 3.70^a^** |
|  |  |  |  |  |  |  |
| **Wash-out Phase** | **2055 ± 386^a^** | **244 ± 42.0^a^** | **82.0 ± 14.0^a^** | **73.0 ± 21.0^a^** | **1.00 ± 0.30^a^** | **8.20 ± 2.70^a^** |
|  |  |  |  |  |  |  |
| **Inter-vention Phase I** | **2260 ± 386^a^** | **224 ± 46.0^a^** | **74.0 ± 18.0^a^** | **109 ± 20.0^b^** | **22.4 ± 0.40^b^** | **11.7 ± 1.70^b^** |
|  |  |  |  |  |  |  |
| **Inter-vention Phase II** | **2388 ± 344^a^** | **235 ± 29.0^a^** | **84.0 ± 20.0^a^** | **111 ± 20.0^b^** | **22.3 ± 0.30^b^** | **11.7 ± 2.10^b^** |

1. Statistical significant differences between the intervention periods were analyzed by one-way ANOVA followed by the Tukey’s post hoc test. Values in a column with different superscript letters represent significant differences (p < 0.05).

**Table S2:** Mean transfer rates (h^-1^) ± SEM calculated for the intervention phases I and II from the individual concentrations of labeled n-3 fatty acids observed in 10 individual volunteers after dietary administration of ^13^C-labeled linseed oil using the tracer model.

| Rate Constants | Phase I | Phase II |
| --- | --- | --- |
| d (3,11) | 0.69 ± 0.04^a^ | 1.11 ± 0.02^b^ |
| d (4,11) | 0.17 ± 0.04^a^ | 0.00 ± 0.02^b^ |
| d (5,11) | 0.09 ± 0.03^a^ | 0.00 ± 0.00^b^ |
| d (6,11) | 0.05 ± 0.02^a^ | 0.01 ± 0.00^b^ |
| k (0,2) | 100 ± 0.00^a^ | 100 ± 0.00^a^ |
| k (0,3) | 0.00 ± 0.00^a^ | 0.00 ± 0.00^a^ |
| k (0,4) | 0.00 ± 0.00^a^ | 0.00 ± 0.00^a^ |
| k (0,5) | 0.00 ± 0.00^a^ | 0.00 ± 0.00^a^ |
| k (0,6) | 0.00 ± 0.00^a^ | 0.00 ± 0.00^a^ |
| k (2,3) | 0.90 ± 0.00^a^ | 0.90 ± 0.00^a^ |
| k (2,4) | 0.00 ± 0.00^a^ | 0.00 ± 0.00^a^ |
| k (2,5) | 0.00 ± 0.00^a^ | 0.00 ± 0.00^a^ |
| k (2,6) | 0.90 ± 0.04^a^ | 0.10 ± 0.01^b^ |
| k (4,3) | 0.42 ± 0.06^a^ | 0.17 ± 0.02^b^ |
| k (5,4) | 0.86 ± 0.08^a^ | 0.08 ± 0.00^b^ |
| k (6,5) | 0.93 ± 0.08^a^ | 0.08 ± 0.01^b^ |
| k (7,1) | 0.20 ± 0.00^a^ | 0.20 ± 0.00^a^ |
| k (8,2) | 0.00 ± 0.00^a^ | 0.01 ± 0.00^b^ |
| k (11,2) | 0.16 ± 0.01^a^ | 0.04 ± 0.01^b^ |
| d (2,7) | 1.00 ± 0.00^a^ | 1.00 ± 0.00^a^ |
| d (3,8) | 1.00 ± 0.00^a^ | 1.00 ± 0.00^a^ |

1. Differences between phase I and II were analyzed by a two-sided, paired t test. Values in a row with different superscript letters represent significant differences (p < 0.05).

**Supplementary figures**

**
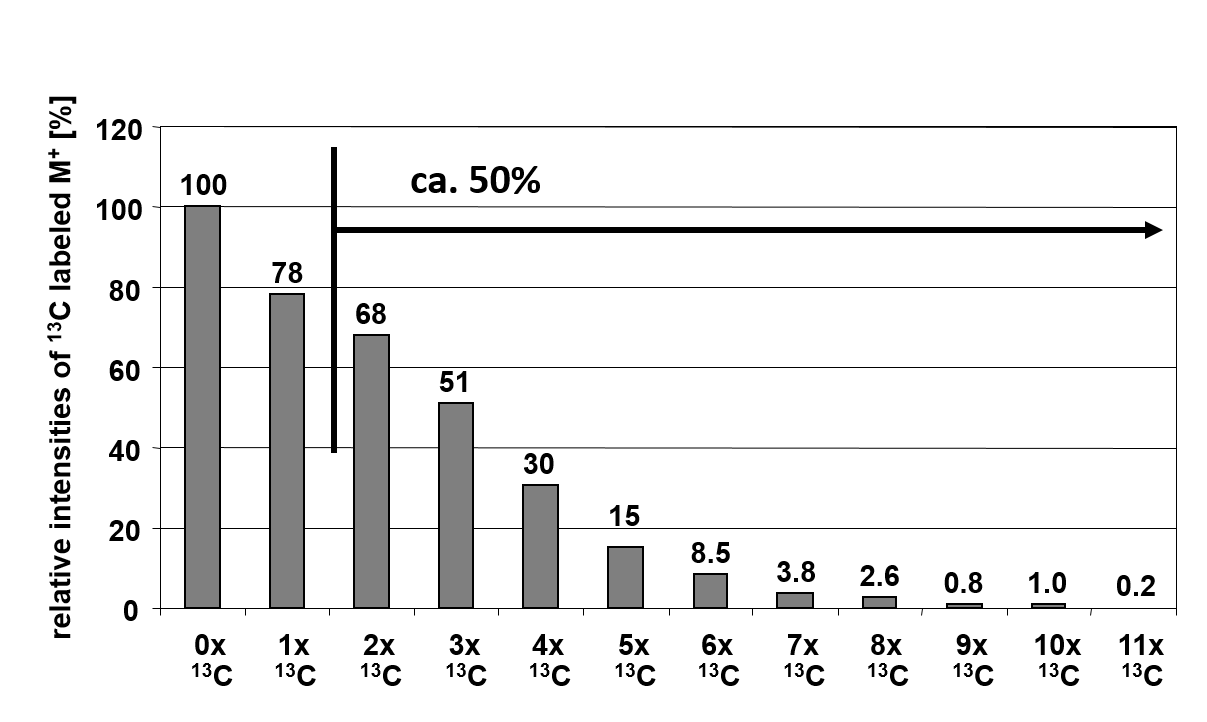
**

**Figure S1.** Proportion of ^13^C-labeling of ALA in flax seeds harvested from flax plants exposed to ^13^C-CO_2_. The x-axis represents the number of ^13^C-labeled atoms in the ALA molecule.
